# Drugging KRAS: current perspectives and state-of-art review

**DOI:** 10.1186/s13045-022-01375-4

**Published:** 2022-10-25

**Authors:** Kaushal Parikh, Giuseppe Banna, Stephen V. Liu, Alex Friedlaender, Aakash Desai, Vivek Subbiah, Alfredo Addeo

**Affiliations:** 1 Mayo Clinic, Rochester, USA; 2 Portsmouth University Hospitals NHS Trust, Portsmouth, UK; 3 Georgetown University, Washington, DC USA; 4 Clinique General Beaulieu, Geneva, Switzerland; 5 MD Anderson Cancer Center, Houston, USA; 6 University Hospital Geneva, Geneva, Switzerland

## Abstract

After decades of efforts, we have recently made progress into targeting KRAS mutations in several malignancies. Known as the ‘holy grail’ of targeted cancer therapies, KRAS is the most frequently mutated oncogene in human malignancies. Under normal conditions, KRAS shuttles between the GDP-bound ‘off’ state and the GTP-bound ‘on’ state. Mutant KRAS is constitutively activated and leads to persistent downstream signaling and oncogenesis. In 2013, improved understanding of KRAS biology and newer drug designing technologies led to the crucial discovery of a cysteine drug-binding pocket in GDP-bound mutant KRAS G12C protein. Covalent inhibitors that block mutant KRAS G12C were successfully developed and sotorasib was the first KRAS G12C inhibitor to be approved, with several more in the pipeline. Simultaneously, effects of KRAS mutations on tumour microenvironment were also discovered, partly owing to the universal use of immune checkpoint inhibitors. In this review, we discuss the discovery, biology, and function of KRAS in human malignancies. We also discuss the relationship between KRAS mutations and the tumour microenvironment, and therapeutic strategies to target KRAS. Finally, we review the current clinical evidence and ongoing clinical trials of novel agents targeting KRAS and shine light on resistance pathways known so far.

## Introduction

Global cancer incidence is on the rise, and the global cancer burden is expected to reach 28.4 million cases in 2040, a 47% rise from 2020 [1]. KRAS is one of the most frequently mutated oncogenes in all human malignancies and is seen in 1 in 7 of all human cancers [2]. KRAS is present and expressed in all human cells as a membrane bound protein.

Understanding and identifying oncogenic driver alterations has changed the management of several cancers, most notably, non-small cell lung cancer (NSCLC) which has emerged as a poster child of precision oncology [3]. Some of the known targetable oncogenic drivers include EGFR mutations, ALK and ROS1 rearrangements, HER2 mutations, MET exon 14 skipping alterations, RET fusions, BRAF V600E mutation, and NTRK fusions. Historically, targeting KRAS mutations has been challenging; it was deemed the “undruggable gene.” Beyond sporadic reports of MEK inhibitors offering transient responses, KRAS has defied all attempts at targeted therapy, clinically [4]. However, concerted efforts in genomic sequencing, therapeutic modeling, drug discovery, medicinal chemistry, pre-clinical validation, and rapid development of the first KRAS G12C inhibitor sotorasib has ushered in a new era of KRAS inhibition. In this review, we explore the historic perspective around KRAS discovery, its normal structure and function, and its role in oncogenesis. We also revisit the past attempts at targeting KRAS mutations and challenges that ensued. The crux of our review focuses on current strategies for targeting specific KRAS mutations as well as strategies to target other related pathways. We conclude with future directions in targeting KRAS and forthcoming advances in the field.

## Discovery of KRAS

**Fig. 1:**
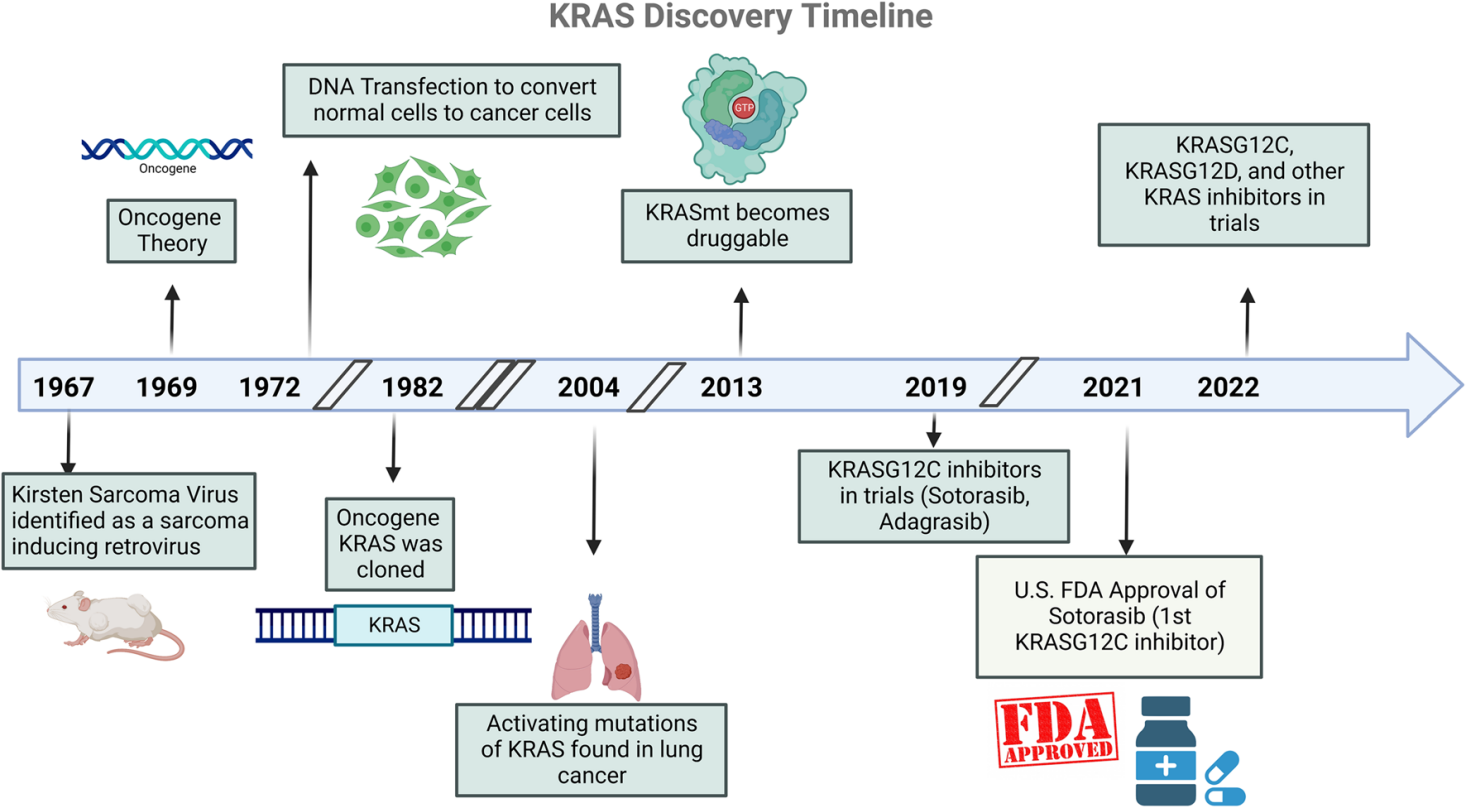
Timeline of KRAS discovery

In 1964, Dr. Jennifer Harvey observed rapid induction of sarcoma in rats infected with Moloney’s leukemogenic virus (MLV) [5]. This was followed by Werner Kirsten’s discovery of similar transformation upon inoculation of a murine erythroblastosis virus in 1967 [6] (Fig. 1). These retroviral transforming genes were named Ha-ras and Ki-ras, respectively, with the term ‘ras’ used as an acronym for “rat sarcoma.” The translational products of v-ras genes were identified in 1979 as a 21 kDa polypeptide, called p21 [7]. Identifying the protein product of ras facilitated in understanding its function, specifically, its high affinity to bind guanine-containing nucleotide [8]. This was a central point in further understanding the function of RAS proteins. Though coined as early as 1969 [9], the term ‘oncogene’ gained traction when these transforming ras genes were identified in human cancers. Their human counterparts, HRAS and KRAS, were discovered in 1982, when human bladder and lung carcinoma cell lines were found to contain DNA sequences homologous to Ha-ras and Ki-ras genes [10]. The following year, the third member of ras family, NRAS, was identified in a neuroblastoma cell line [11]. In 1984, an activating KRAS G12R point mutation was first identified in the tumour tissue of a 66-year-old man with squamous cell lung carcinoma, which was not present in normal parenchymal or lymphocytes [12]. KRAS G12C mutation was identified in the same year in a human lung tumour (PR371) that was propagated in nude mice [13]. These were some of the earliest studies to show that malignant activation of KRAS oncogene was specifically associated with human carcinogenesis.

## Biology of KRAS

**Fig. 2:**
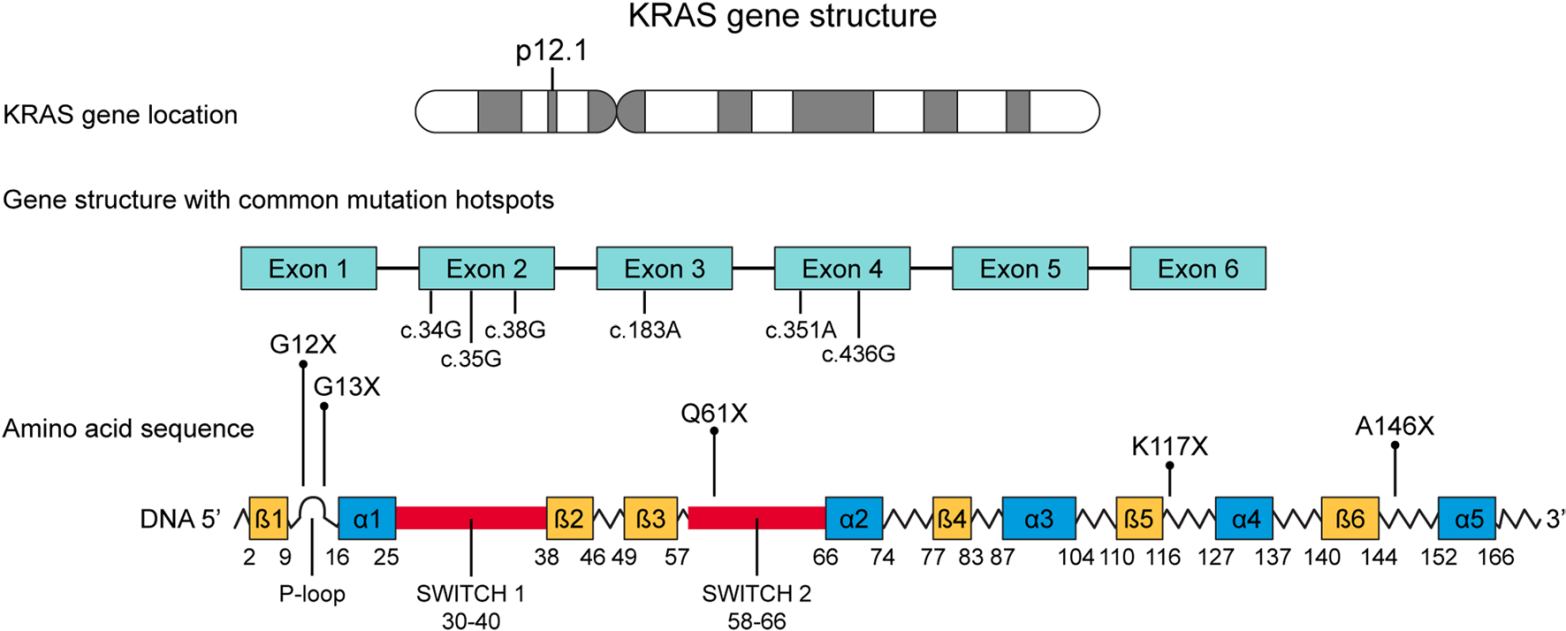
KRAS gene and mutational hotspots

KRAS has been identified as a KRAS-1 pseudogene on short arm of chromosome 6 and KRAS-2 gene, located on the short arm of chromosome 12 (12p11.1-12p12.1) [14]. KRAS-2 coding region spans across six exons and measures over 45 kB. The two protein isoforms of KRAS-2, KRAS-4A and KRAS-4B are produced due to alternative splicing on its fourth exon, leading to 188 and 189 monomeric amino acid sequences, respectively [15]. For clinical and research purposes, the term KRAS refers to KRAS-4B, which constitutes the major transcriptomic product in human cells. RAS proteins belong to the super family of small GTPases and bind exclusively to GTP (G proteins) [16]. KRAS protein product consists of 2 domains, the N-terminal catalytic (guanine binding) domain (G-domain) and the hypervariable region (HVR) at the C-terminal (Fig. 2). The catalytic domain is a highly conserved region with a high degree of homology [17]. It consists of the P-loop, switch I, and switch II regions. The G-domain facilitates GTP-GDP exchange and functions as a GTP-GDP switch [18]. The P-loop is the phosphate binding region and stabilizes the nucleotide phosphates while the switch regions form binding surfaces for effector proteins. This G-domain switch is regulated primarily by guanine exchange factors (GEFs) that promote GDP to GTP switch and activation, and deactivating factors such as GTPase activating proteins (GAPs).

The C-domain has a high degree of variability and is responsible for anchorage of RAS to the inner surface of plasma membrane. It includes the CAAX box (cysteine, 2 aliphatic amino acids, another residue) and is responsible for post-translation modifications such as prenylation. Prenylation is a process by which a farnesyl or geranylgeranyl moiety is added to the terminal cysteine of the CAAX box by farnesyltransferase (FTase) or geranylgeranyltransferase (GGTase) [19]. This is followed by cleavage of the AAX residues and methylation of the cysteine residue by isoprenylcysteine methyltransferase (ICMT) [20]. KRAS monomers require localization to the cell membrane for their activity. More recent evidence suggests that KRAS monomers undergo dimerization for their downstream signaling activity [21].

KRAS activation can occur because of several upstream signals such as growth factors like epidermal growth factor (EGF), platelet derived growth factor (PDGF), insulin-like growth factor (IGF), and fibroblast growth factor (FGF), receptor tyrosine kinase (RTK) activation, and cytokines. KRAS activation consists of phosphorylation of GDP-bound KRAS to a GTP-bound state with the assistance of RAS GEFs. CDC25 was the first RAS GEF identified in S. cerevisiae [22], followed by the discovery of homologous son of sevenless (SOS) gene in drosophila [23]. Mammalian counterparts of CDC25, RAS guanine-nucleotide-releasing-factor (RASGrf) and son of sevenless (SOS) proteins were later identified [24, 25]. When EGF binds to its receptor (EGFR), it leads to dimerization and phosphorylation of EGFR by tyrosine kinase activation. The phosphorylated-EGFR dimer then binds to the sequence homology 2 (SH2) domain of growth factor receptor bound protein 2 (GRB2). EGFR-GRB2 complex binds to SOS via its SH3 domain and leads to membrane localization of SOS [26–29]. SOS1 and SOS2 are GEFs that promote decoupling of GDP from RAS and facilitate GTP-RAS interaction. GTP-RAS binding leads to a conformational change in the Switch I and II regions and promotes downstream signaling [30]. A second tyrosine phosphatase enzyme, Src homology phosphatase 2 (SHP2) also activates KRAS using mechanisms that are not completely elucidated [31]. Current evidence suggests that SHP2 functions as a scaffolding protein and enhances GRB2-SOS1 binding and promotes KRAS activation [32]. PTPN11 gene, which codes for SHP2, can be mutated in patients with Noonan Syndrome, an autosomal dominant RASopathy, characterized by cardiac, endocrine, neurodevelopmental and hematologic disorders [33].

KRAS inactivation also requires a complex interplay of multiple molecules. After activation, the RAS-GTP complex undergoes intrinsic GTP hydrolysis to its inactive RAS-GDP state. Many GAPs further accelerate GTP hydrolysis manifold and lead to RAS deactivation. GAP-mediated hydrolysis is the dominant mechanism of RAS-GTP hydrolysis. The most prominent RAS GAPs are neurofibromin 1 (NF1) and p120GAP. Oncogenic KRAS mutations lead to decreased intrinsic GTPase activity and significantly increased resistance to GAP-mediated hydrolysis [34]. Similarly, mutations in RAS GAPs prevent GTP hydrolysis and cause persistent RAS activation, downstream signaling, and eventual carcinogenesis. Germline mutations in NF1 gene are associated with neurofibromatosis, central nervous system neoplasms, sarcomas, and leukemias.

## KRAS-mediated signaling pathways

**Fig. 3:**
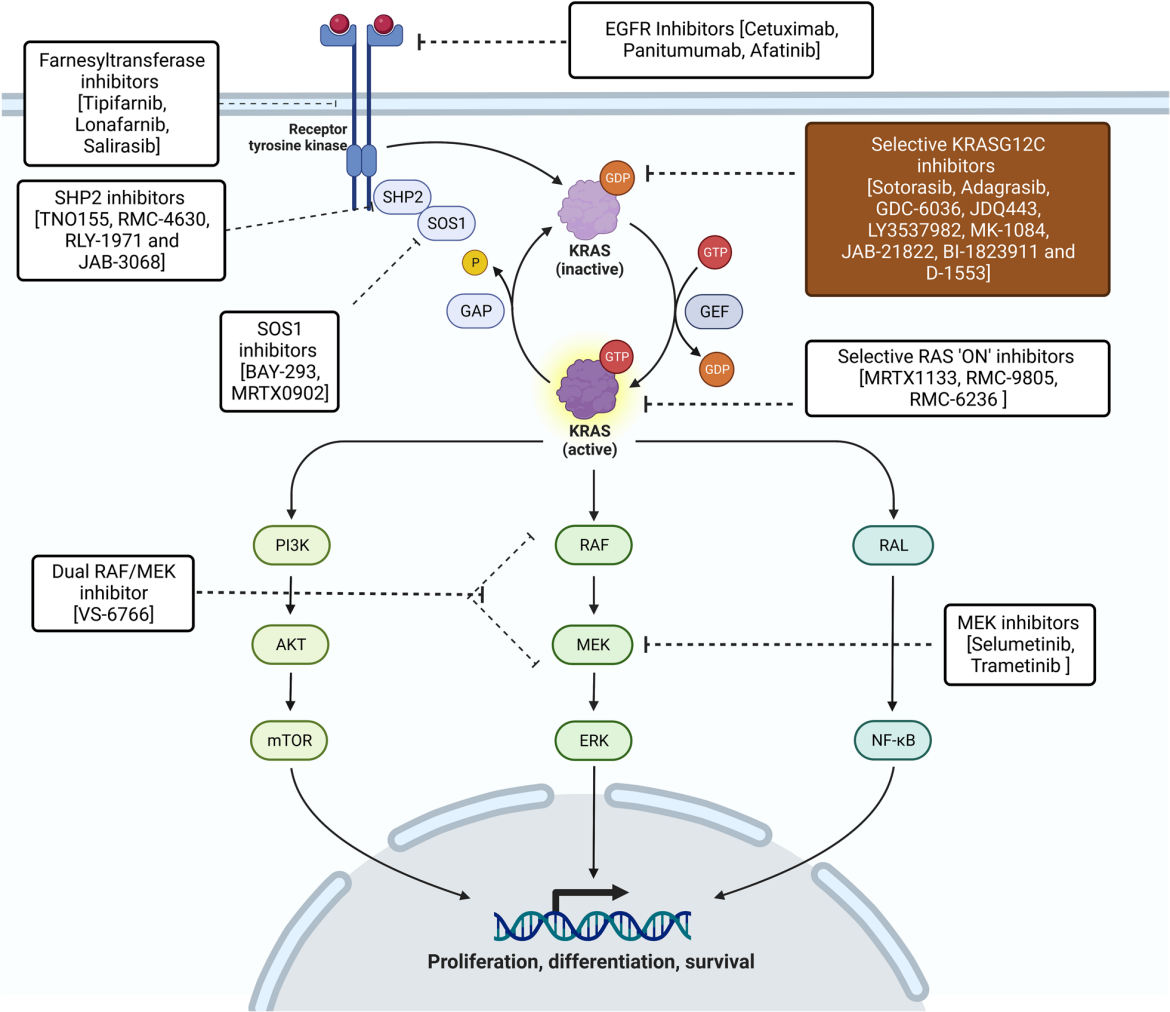
KRAS signaling pathways with targeting drugs

KRAS activation leads to downstream signaling of three major pathways: the MAP kinase pathway, PI3K-AKT-mTOR pathway, and the tumour invasion and metastasis-inducing protein 1 (TIAM1-RAC) and RAS-related protein (RAL) pathways (Fig. 3). The MAPK pathway consists of RAS, RAF, MEK and ERK phosphorylation and regulates cell-cycle and cellular proliferation. RAS activation and dimerization leads to conformational changes that allows binding and phosphorylation of RAF molecules. In the case of mutant RAS, its dimers allow for increased RAF binding and activation [35]. This constitutes the major downstream signaling pathway of mutant RAS. The final enzyme in the MAPK pathway, ERK translocates to the nucleus and activates various transcription factors [36]. This promotes cellular proliferation and differentiation.

Phosphatidylinositol-4,5-bisphosphate 3-kinase (PI3K) activation by GTP-RAS complex leads to phosphorylation of phosphatidylinositol 4,5-biphosphate (PIP2) to phosphatidylinositol 3,4,5-triphosphate (PIP3) [37]. PIP3 then phosphorylates AKT, which in turn causes downstream mTOR, FOXO and NF-kB phosphorylation, leading to cell survival and resistance to apoptosis [38]. The PI3K pathway is frequently upregulated in RAS mutations; however, the role of RAS on PI3K activation in normal cells remains unclear.

RAL guanine nucleotide stimulator (RALGDS) is another downstream protein activated by RAS, that promotes cell migration and RAS-dependent tumour growth [39]. RALGDS also stimulates Jun N-terminal kinase (JNK) pathway, which leads to transcription and cell-cycle progression. TIAM1-RAC are GEFs that lead to phosphorylation of PAK serine/threonine kinases, which is involved in cytoskeleton rearrangement and cell migration [40].

Thus, KRAS in its normal state is responsible as a key link between several cell-cycle pathways and an activating KRAS mutation leads to oncogenesis by multiple downstream activation pathways.

## Oncogenic KRAS mutations

**Fig. 4:**
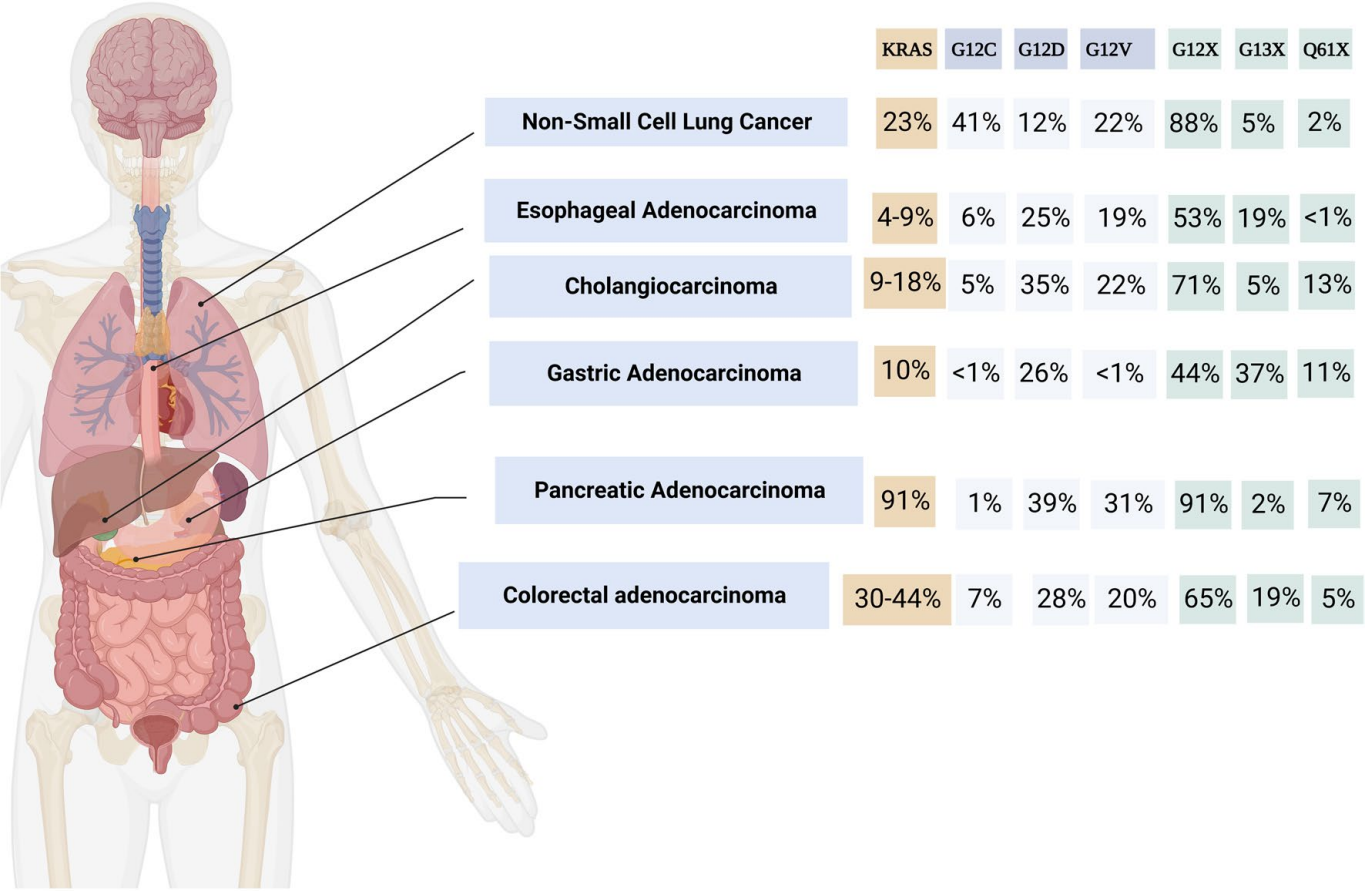
KRAS incidence across aerodigestive malignancies

KRAS mutations are seen in a variety of malignancies at different rates. Its incidence is highest in pancreatic cancers (> 85%) followed by colorectal cancer (~ 40%), NSCLC (~ 30%) and cholangiocarcinoma (~ 20%) (Table 1, Fig. 4). KRAS mutations are often early events in tumourigenesis in lung and pancreatic cancers [41, 42]. In contrast, KRAS mutations are usually progression events in colon cancers that follow a driver mutation in the Wnt signaling pathway [43]. An overwhelming majority of KRAS mutations are single-base missense mutations, found commonly at codons 12 (83%), 13 (14%), or 61 (2%) on exons 2 and 3. Codons 12 and 13 are located in the P-loop coding region while codon 61 is located in the switch II coding region [44]. Codons 59, 117, and 146 are also commonly found mutated in multiple malignancies. Missense mutations in these codons lead to increased GTP binding, KRAS activation, and downstream signaling through a variety of mechanisms. For instance, a G12C missense mutation represents a single nucleotide substitution at c.34G > T, coding for the amino acid cysteine instead of glycine. This leads to a conformational change at the GAP binding site and inhibits GAP-mediated GTP hydrolysis. Oncogenic KRAS mutations in codons 12, 13, and 61 inhibit GTP hydrolysis and prevent KRAS deactivation. Apart from GAP-mediated hydrolysis, oncogenic KRAS mutations may also affect rates of intrinsic GTP-hydrolysis. KRAS G12A, G12R, Q61H, and Q61L mutations were found to have 40–80 fold decrease in intrinsic hydrolysis; G12V, G12D, and G13D mutations had an intermediate reduction in intrinsic hydrolysis [44]. Interestingly, while other mutations inhibit intrinsic KRAS GTPase activity, G12C mutation exhibits wild-type intrinsic GTPase activity, allowing it to somewhat shuttle between GDP and GTP bound states. p120GaP-mediated GTP hydrolysis is severely restricted in all KRAS mutations. Codon 13 mutations can exhibit intermediate sensitivity to NF1-mediated hydrolysis while codons 12 and 61 mutations are insensitive to NF1-mediated hydrolysis [45]. KRAS mutations have been shown to activate distinct signaling pathways. G12A, G13D and Q61L mutations have a high affinity to RAF and favorably activate the MAPK pathway [44]. G12C and G12V mutations are shown to preferentially activate RAL signaling and G12D-mutant cell lines are shown to have high levels of phosphorylated AKT, suggesting preferential activation of PI3K-AKT-mTOR pathway [46, 47].

**Table 1:** KRAS mutation incidence across aerodigestive malignancies

Aerodigestive malignancy	KRAS mutation incidence (%)	G12C (%)	G12D (%)	G12V (%)	G12X (%)	G13X (%)	Q61X (%)
Pancreatic adenocarcinoma	91 [173]	1	39	31	91	2	7
Non-small cell lung cancer	23 [174]	41	12	22	88	5	2
Adenocarcinoma	33 [175]						
Squamous cell carcinoma	5 [176]						
Colorectal adenocarcinoma	27.9 [177]–43.7 [178]	6.5	27.5	20	65	19	4.5
Cholangiocarcinoma	9.5 [179]–18.2 [180]	5	35	22	71	5	13
Esophageal carcinoma	4.5 [181]–9.1 [182]	6	25	19	53	19	< 1
Gastric adenocarcinoma	9.8 [183]	< 1	26	< 1	44	37	11

Data extracted using www.cBioPortal.org

KRAS G12C is the commonest KRAS mutation in NSCLC, seen in about 14% of all lung adenocarcinomas, followed by G12V. The spectrum of KRAS mutations in lung cancer is heterogenous. While G12C is the commonest KRAS mutation among smokers (44%), followed by G12V (19%), G12D is the most frequent mutation in never-smokers (56%) [48]. Intriguingly, G12C was more commonly mutated in women than other KRAS mutations despite lower tobacco exposure than men, suggesting increased susceptibility to acquiring smoking-dependent G12C mutation in women. In another retrospective analysis of 2327 patients with KRAS-mutant NSCLC, KRAS G12D mutation was enriched in never-smokers (22%) or lower pack-year smoking history (median 22.5 pack years) [49]. In pancreatic cancers, KRAS G12D and G12V are the predominant mutations, at 40% and 32% of all KRAS mutations, respectively, while G12R mutation accounts for nearly 17% of all KRAS mutations in pancreatic cancer [50]. Right-sided colorectal tumours have a predilection for KRAS mutations and G12 mutations make up about 65% of all KRAS mutations. Like in pancreatic cancers, G12D and G12V are most frequent mutations. G13 mutations are also seen in about 18% of all KRAS mutations in colon cancers but are almost never seen in lung and pancreatic cancers. Notably, mutations in codons 117 (K117N/R) and 146 (A146T/V) are seen exclusively in colon cancers [51].

## Co-mutational landscape

KRAS mutations often occur with specific co-mutations that impact the function of KRAS and oncogenesis. For instance, mutations in codon 13 often co-occur with NF1 mutations, limiting NF1-mediated hydrolysis [45]. In a dataset of 1078 patients with KRAS-mutant NSCLC, 577 (53.5%) patients had at least one additional co-mutation [52]. 141 (24.4%) of patients had two co-mutations. Mutations in TP53 were present in 39.3%, followed by STK11 (19.8%), KEAP1 (12.9%) and ATM (11.9%). Mutations in serine threonine kinase 11 (STK11 or LKB1) tumour suppressor gene are seen in about 10% of patients NSCLC. Depending on the specific KRAS mutation, frequency of STK11 mutations ranged from 14.2% (G12D) to 39.2% (G13) suggesting a pathologic co-occurrence [53]. 23% of patients with KRAS G12C mutations also had STK11 co-mutation [53]. STK11 co-mutations have been associated with worse survival in KRAS-mutant lung cancers [54, 55]. In one study, median OS for KRAS-mutant NSCLC was 21 months whereas it was 12 months for KRAS/STK11 double-mutant NSCLC (HR 1.7, 95% CI 1.1–2.4, *p* = 0.002) [56]. Similarly, KEAP1 or NFE2L2 co-mutations with KRAS have also been associated with poor outcomes. In the same analysis, patients with KEAP1 or NFE2L2 co-mutation had a median OS of 10 months (HR 2.1, 95% CI 1.4–3.1, *p* < 0.0001). Unexpectedly, p53 co-mutations were not prognostic or predictive in multiple analyses [56–58]. Of note, de novo KRAS mutations are thought to be mutually exclusive to EGFR, ALK, and BRAF mutations. In the cases of these mutations, KRAS mutations can develop as resistance mutations under pressure from targeted therapies [59–61].

In colon cancer, APC mutations, often the first event in oncogenesis, accompany KRAS mutations in over 80% of cases, followed by p53 (55%) and PIK3CA (33%) co-mutations [62]. In pancreatic cancer, KRAS mutation is often the initiating genetic event leading to pancreatic intraepithelial neoplasms. Carcinogenesis and metastasis are stimulated by acquiring other mutations such as p53 (64%), SMAD4 (21%), and CDKN2A (17%) [63].

## Tumour microenvironment (TME) and immune response

Immune cells and inflammatory products play a pivotal role in carcinogenesis and activity of immunotherapy. The tumour microenvironment acts as a stage for the interplay between anti-tumour-immune response cells such as CD8 + T cells, NK cells, CD4 + Helper T cells and M1 macrophages and immunosuppressive cells such as myeloid-derived suppressor cells (MDSCs) and regulatory T cells (Tregs). KRAS mutations regulate the tumour microenvironment and alter tumour stroma, immune cell infiltration and cytokine expression. Mutant KRAS induces inflammatory cytokines such as CXCL-8, IL-1, and NF-κB [64]. These cytokines promote tumourigenesis by increasing tumour vascularity, tumour invasiveness, stromal remodeling, and immune suppression. The high prevalence of KRAS mutations is thought to play an important role in the universally immunosuppressive TME on pancreatic adenocarcinoma. KRAS mutations activate Yes-associated protein-tafazzin (YAP-TAZ) and Janus kinase-signal transducers and activators of transcription (JAK-STAT) pathways, leading to release of immunosuppressive cytokines such as CSF-1, IL-4, IL-6. These cytokines recruit a variety of immunosuppressive cells such as cancer associated fibroblasts, myeloid-derived suppressive cells, tumour associated macrophages, and regulatory T cells [65–67]. Consequently, immune checkpoint inhibitors (ICIs), thus far, have shown negligible activity in pancreatic adenocarcinoma (PDAC). In a phase II randomized study, durvalumab alone or in combination with tremelimumab showed an objective response rate (ORR) of 0% and 3.1% in second or later-line treatment of PDAC [68]. In colon cancer, mutant KRAS has been shown to inhibit expression of interferon regulatory factor2 (IRF2), which is required for interferon-mediated immune responses [69]. Liu et al. analyzed the tumour immune landscape 528 patients with colon cancer (224 KRAS_mt_ and 304 KRAS_wt_) and observed downregulation of immunoactive cells such as native B cells, M1 macrophages and CD4 memory T cells and abundance of immunosuppressive Tregs in KRAS_mt_ tumours [70]. In NSCLC, KRAS G12D mutation has been associated with a lower tumour-mutation burden, decreased PDL1 expression, and reduced CD8 + T cell infiltration in the TME [49]. Compared to non-G12D mutations, KRAS G12D mutations are also associated with poorer overall survival with immune checkpoint inhibition (HR 1.45, 95% CI 1.05–1.99; *p* = 0.02). Inhibition of KRAS G12C was also shown to promote an inflammatory TME by recruiting CD8 + T-cells and antigen presenting dendritic cells, and by increasing interferon signaling and chemokine production [71].

KRAS co-mutation status may also impact the tumour microenvironment and the effect of immune checkpoint inhibition. KRAS/STK11 co-mutations often have a CD8 + T-lymphocyte deficient and T-regulatory cell rich microenvironment whereas tumours with KRAS/p53 co-mutations have an inflamed tumour microenvironment, rich in CD8 + T-lymphocytes [54, 56]. This can be attributed to the propensity of p53 mutations to increase somatic tumour mutations that potentially induce tumour neoantigen development. Using genomic and transcriptomic analyses, Skoulidis et al. [72] grouped KRAS mutations into three clusters, based on their co-mutational profile: KP cluster for KRAS_mt_/p53_mt_, KL cluster for KRAS_mt_/STK11_mt_, and KC cluster for KRAS_mt_/CDKN2A/B_mt_, with each cluster showing biological heterogeneity. The KL cluster, for instance had a pauci-immune tumour microenvironment and these tumours tended to have low PD-L1 expression. The KC cluster, on the other hand, expressed mucinous histology and suppressed mTORC1 signaling, and tumours were frequently TTF1 negative. They subsequently showed that efficacy of anti-PD(L)1 inhibitors differed significantly between these groups, with objective responses for KL, KP, and K-only subgroups being 7.4%, 35.7%, and 28.6%, respectively (*p* < 0.001). Progression-free survival (PFS) and overall survival (OS) were also poor for patients in the KL group compared to or K-only groups [73]. In another recent combined cohort of 536 patients with KRAS-mutant lung adenocarcinoma, both STK11 and KEAP1 mutations in the presence of a KRAS mutation were associated with poor response rates to anti-PD(L)1 inhibitors [74]. Median PFS and OS for KRAS_mt_/STK11_mt_ NSCLC were 2.0 and 6.2 months, respectively, whereas those for KRAS_mt_/STK11_wt_ were 4.8 (HR 2.04, 95% CI 1.66–2.51, *p* < 0.0001) and 17.3 months (HR 2.09, 95% CI 1.68–2.61 *p* < 0.0001), respectively. For KRAS_mt_/KEAP1_mt_, PFS and OS were 1.8 and 4.8 months, respectively, while for KRAS_mt_/KEAP1_wt_, they were 4.6 (HR 2.05, 95% CI 1.63–2.59, *p* < 0.0001) and 18.4 months (HR 2.24, 95% CI 1.74–2.88, *p* < 0.0001), respectively.

In summary, KRAS mutations independently, and in conjunction with other co-mutations influence tumour growth, changes in tumour microenvironment, and the efficacy of immunotherapy.

## Targeting KRAS mutations

Historically, targeting KRAS mutations had been unsuccessful due to three major reasons. The RAS molecule has a high affinity in picomolar range to abundantly available cytoplasmic GTP making competitive inhibition challenging. Secondly, unlike other molecules with a targeted inhibitor, the RAS protein has a smooth surface without expression of a drug binding groove or pocket. Lastly, RAS pathways have several upstream and downstream regulators, which allow for multiple resistance mechanisms and bypass signals to overcome inhibition [75]. The development of resistance via multiple pathways suggests that some combination strategies may not be fruitful in targeting drug resistance.

## Inhibition of KRAS membrane localization

Tipifarnib and lonafarnib, both farnesyltransferase inhibitors (FTIs) generated early excitement in mouse models and early phase trials [76–78]. These agents inhibit the prenylation and plasma membrane localization of RAS by inhibiting a key enzyme, farnesyltransferase. Since membrane localization of KRAS is essential for its downstream signaling, blocking it appeared a sound rationale for inhibition of mutant KRAS. However, preclinical excitement did not transcribe to clinical utility with multiple negative phase II and phase III studies, likely due to presence of bypass prenylation pathway by geranylgeranylation [79]. For instance, in a phase II study of tipifarnib in 44 patients with advanced NSCLC, no objective responses were observed [80]. Of note, HRAS selectively relies on farnesylation for plasma membrane localization and FTIs are being evaluated in HRAS-mutant head and neck malignancies. A phase II study of tipifarnib in HRAS-mutant head and neck squamous cell carcinoma showed an objective response rate of 55%, PFS of 5.6 months, and median OS of 15.4 months [81]. Interestingly, lonafarnib was approved in the US for treatment of Hutchinson-Gilford Progeria Syndrome (HGPS), where inhibition of farnesylation limits accumulation of progerin and progerin-like proteins in the nucleus and cellular cytoskeleton [82]. Salirasib, a second-generation agent that inhibits membrane localization of all activated RAS isoforms (KRAS, NRAS, and HRAS), also failed to show any meaningful activity in a phase II clinical trial, wherein none of 33 patients had any response [83]. Further development of this agent was halted.

## Selective inhibition of mutant KRAS

Pioneering efforts in targeting mutant KRAS came from the lab of Dr. Kevan Shokat. In 2013, Ostrem et al. [84] identified an allosteric binding site behind the switch II region in KRAS G12C-mutant protein. This switch II pocket was only seen in G12C-mutant protein, making it a target for drug development. Cysteine is the most reactive amino acid, and experiences with agents such as ibrutinib and dacomitinib have shown that active cysteine acts as a nucleophile to form covalent bonds with these agents [85, 86]. Ostrem et al. developed multiple covalent inhibitors, which bind to the cysteine residue at switch-II pocket (S-IIP) of the KRAS-GDP complex. This leads to a conformational change at switch I and switch II regions, preventing its activation and downstream signaling, and eventually inducing cell apoptosis. Notably, these compounds did not block the GTP-bound state and require intrinsic GTPase hydrolysis of KRAS G12C to the GDP-bound state for activity. Compounds 6 and 12 were the most potent in-vitro covalent inhibitors developed by Ostrem and colleagues, however, these lacked cellular activity.

This discovery heralded a new frontier in drug discovery and led to the development of clinically active agents. The first prototypes were SML-8-73-1, ARS-853 and ARS-1620. SM-8-73-1 was designed as a guanosine-derived GDP analogue that binds to KRAS G12C, however, this compound lacked cellular penetrance [87, 88]. On the other hand, compounds such as ARS-853 and ARS-1620 target the allosteric site at the S-IIP and inactivate KRAS G12C by trapping it in a GDP-bound state. ARS-853 was found to be 600-fold more potent than compound 12 developed by Ostrem et al. in engaging KRAS G12C and led to a dose-dependent inhibition of SOS catalyzed nucleotide exchange and downstream MAPK signaling in KRAS G12C cells [89]. To overcome its inherently poor metabolic and chemical stability, quinazoline-based ARS-1620 was developed as an oral, selective, G12C inhibitor that interacts with His-95 providing a more rigid and favorable conformation that ARS-853 [90]. Further clinical development required additional ligand interactions that were limited by small size of S-IIP. Using the His-95 groove as an additional binding site, AMG-510 and MRTX849 were the first KRAS G12C inhibitors that were introduced in clinic [71, 91, 92]. The introduction and eventual success of these molecules have generated great excitement in targeting what was once an ‘undruggable’ mutation. Other KRAS G12C inhibitors in development are GDC-6036, JDQ443, LY3537982, MK-1084, JAB-21822, BI-1823911 and D-1553.

### KRAS G12C inhibitors

**Fig. 5:**
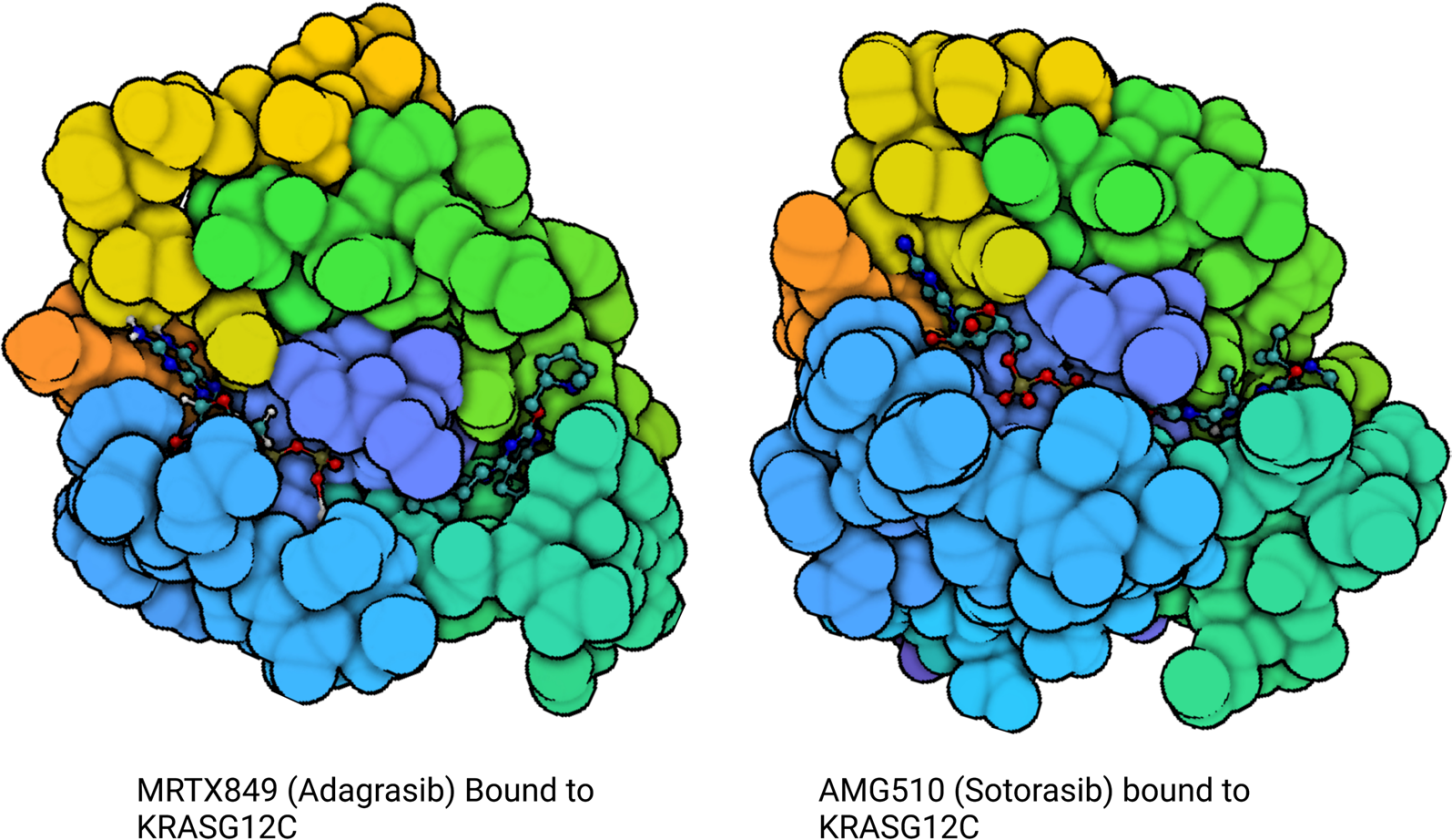
KRAS G12C structure with S-IIP inhibitor in place

The development of selective KRAS G12C inhibitors, led by sotorasib (AMG 510) and adagrasib (MRTX849), had immediate and profound clinical impact. Both sotorasib and adagrasib are orally bioavailable small molecules that selectively and irreversibly bind KRAS G12C in its GDP-bound state, locking it in its inactive conformation (Fig. 5). Early clinical studies explored efficacy across tumour types and while responses were observed in different cancers, monotherapy was most effective in NSCLC.

The phase I/II CodeBreaK100 trial explored sotorasib monotherapy in tumours harbouring a KRAS G12C mutation [93]. In the phase I dose escalation portion, no dose-limiting toxicities were encountered at the planned dose levels of 180 mg, 360 mg, 720 mg, and 960 mg, with 960 mg established as the recommended phase II dose. In the 129 patients treated across dose levels, treatment-related adverse events (TRAE) were observed in 56.6% of patients but only 11.6% of patients experienced grade 3 or 4 TRAE. The most common grade 3 or higher treatment related adverse events (TRAEs) were elevations in ALT (4.7%) and AST (2.3%), anemia (4.7%), vomiting (3.9%), diarrhea (3.9%) and abdominal pain (3.1%). With a median follow-up of 11.7 months, the response rate among patients with KRAS G12C NSCLC (*n* = 59) was 32.2% with a disease control rate of 88.1%. The phase II dose of 960 mg was given to 34 patients with NSCLC and the response rate in that cohort was 35.3%. Responses were rapid, with a median time to response (TTR) of 1.4 months, and durable, with a median duration of response (DOR) of 10.9 months. The median PFS was 6.3 months for all patients with NSCLC. The phase I portion also included 42 patients with colorectal cancer, where the response rate was only 7.1%, though 73.8% of patients achieved stable disease with a median duration of stable disease of 5.4 months.

The phase II portion of CodeBreaK100 explored sotorasib 960 mg once daily in cancers with a KRAS G12C mutation [94]. The NSCLC cohort included 126 patients, 81% of whom had received prior platinum-based chemotherapy and PD(L)1 inhibitor therapy. With a median follow-up of 15.3 months, the response rate was 37.1%; 80.6% of patients achieved disease control. Response characteristics were consistent with the phase I portion, with a median time to response of 1.4 months and a median duration of response was 11.1 months. Median PFS was 6.8 months and median survival was 12.5 months (Table 2). The safety profile of sotorasib in the phase II portion was consistent with that seen in the phase I portion. The most common TRAEs included diarrhea (31.7% all grade, 4% grade 3), nausea (19.0% all grade, 0% grade 3+), increase in ALT (15.1% all grade, 6.3% grade 3), increase in AST (15.1% all grade, 5.6% grade 3), and fatigue (11.1% all grade, 0% grade 3+). TRAE necessitated dose reduction and/or modification in 22.2% and discontinuation in 7.1% of patients. The US FDA granted accelerated approval to sotorasib for patients with KRAS G12C-mutant NSCLC with at least one prior line of systemic therapy on May 28, 2021 [95]. CodeBreaK200 is the first randomized phase III trial of sotorasib to report study results [96]. This trial randomized 345 patients with previously treated metastatic KRAS G12C-mutant NSCLC, who had not received prior KRAS G12C inhibitors to receive sotorasib (*n* = 171) or docetaxel (*n* = 174). Patients were allowed to crossover from docetaxel to sotorasib upon disease progression. The primary endpoint of this trial was progression free survival. The trial met its primary endpoint and PFS was 5.6 months (95% CI 4.3–7.8) with sotorasib and 4.5 months (95% CI 3.0–5.7) with docetaxel (HR 0.66, 95% CI 0.51–0.86; *p* = 0.002). 12-month PFS was 24.8% with sotorasib and it was 10.1% for docetaxel. Secondary endpoints for this trial are OS, ORR, DOR and TTR. ORR was 28.1% for sotorasib and 13.2% for docetaxel. Median DOR and TTR were superior for sotorasib at 8.6 and 1.4 months, respectively, while for docetaxel, they were 6.8 and 2.8 months, respectively. However, the study did not meet its secondary endpoint of OS. Median OS was 10.6 months (95% CI 8.9–14.0) for sotorasib and 11.3 months (9.0–14.9) for docetaxel (HR 1.01, 95% CI 0.77–1.33; *p* = 0.53). While the OS data are not yet mature and the study was underpowered to show OS differences, these results are disappointing and highlight the challenges with targeting KRAS.

**Table 2:** Efficacy results from single arm phase II studies of sotorasib (CodeBreaK100 [94]) and adagrasib (KRYSTAL-1 [100]) in NSCLC

	Evaluable KRAS G12C NSCLC	Response rate 9) (%)	Median time to response (months)	Median duration of response (months)	Median PFS (months)	Median survival (months)
Sotorasib 920 mg daily	124	37.1	1.4	11.1	6.8	12.5
Adagrasib 600 mg bid	112	42.9	1.4	8.5	6.5	12.6

Efficacy of sotorasib monotherapy in other KRAS G12C-mutant colorectal cancers was less impactful. The phase II portion of CodeBreak100 for KRAS G12C-mutant colorectal cancer included 62 patients with prior fluoropyrimidine, oxaliplatin, and irinotecan treatment [97]. The ORR was only 9.7% (*n* = 6), and though 82.3% achieved disease control, the overall median PFS was only 4.0 months. In 38 patients with pancreatic cancer, sotorasib led to an ORR of 21.1% and DCR of 84.2% [98].

Adagrasib monotherapy was studied in the phase I/II KRYSTAL-1 trial. The phase I portion utilized an accelerated titration design to explore doses of 150 mg daily, 300 mg daily, 600 mg daily, 1200 mg daily, and 600 mg twice daily [99]. As with the CodeBreaK100 trial, inclusion for KRYSTAL-1 was limited to tumours harbouring a KRAS G12C mutation. Of the 25 patients included, 18 (72%) had NSCLC. The median number of prior therapies was 3. No maximally tolerated dose was identified, though five patients had at least one dose limiting toxicity; dose limiting toxicities included diarrhea, nausea, fatigue, amylase elevation, lipase elevation, and decreased appetite. The 600 mg twice daily dose of adagrasib was recommended for phase II trials. At the 600 mg bid dose level, TRAEs included nausea (80% all grade, no grade 3+), diarrhea (70% all grade, no grade 3+), vomiting (50% all grade, no grade 3+), and fatigue (45% all grade, 15% grade 3). There was one grade 5 TRAE in the phase I portion: pneumonitis in a patient with underlying pneumonitis from prior therapy. Dose reduction was required in 65% of patients but the median relative dose intensity was over 90%. There were 15 evaluable patients with KRAS G12C NSCLC treated with adagrasib 600 mg bid in the phase I portion of KRYSTAL-1. In this cohort, the ORR was 53.3% with a median duration of response of 16.4 months and a median PFS of 11.1 months. Median survival was not yet reached but the 12-month survival rate was 66.7%.

The phase II portion of KRYSTAL-2 included 116 patients with KRAS G12C NSCLC treated with adagrasib 600 mg bid [100]. All patients had prior platinum-doublet chemotherapy and 98.3% had received both chemotherapy and checkpoint inhibitor therapy. With a median follow-up of 12.9 months, the ORR was 42.9%. The time to response was 1.4 months and the median duration of response was 8.5 months. Median PFS was 6.5 months; with extended follow-up to 15.6 months, the median survival was 12.6 months (Table 2). TRAEs included diarrhea (62.9% all grade, 0.9% grade 3), nausea (62.1% all grade, 4.3% grade 3), vomiting (47.4% all grade, 0.9% grade 3), fatigue (40.5% all grade, 6.9% grade 3), increase in ALT (27.6% all grade, 5.2% grade 3), increase in creatinine (25.9% all grade, 0.9% grade 3), and increase in AST (25.0% all grade, 5.2% grade 3). TRAEs led to dose reduction in 51.7% and dose interruption in 61.2% but only 6.9% of patients discontinued adagrasib due to TRAE.

Adagrasib has shown efficacy in patients with KRAS G12C NSCLC with treated and untreated brain metastases. Among 33 patients with previously treated, stable central nervous system metastases, the intracranial confirmed objective response rate was 33.3% (95% CI, 18.0–51.8) [100]. In a report of 2 patients with untreated brain metastases enrolled on the KRYSTAL-1 phase IB cohort, one patient had resolution of 3 untreated brain metastases and the other had a decrease in size of 3 baseline brain metastases [101]. Cerebrospinal fluid analysis showed adagrasib concentrations of 24.2–34.6 nM. These results mirror those seen in preclinical mouse models; adagrasib has also demonstrated efficacy in intracranial xenograft mouse models.

Adagrasib is also being studied in other KRAS G12C cancers. In a report of 46 patients with KRAS G12C colorectal cancer, the ORR with adagrasib was an encouraging 22% with a disease control rate of 87% [102]. The median duration of response was 4.2 months with a median PFS of 5.6 months.

The development of sotorasib and adagrasib has expanded potential treatment options for patients with KRAS G12C NSCLC. A greater understanding of the mutational landscape of these cancers and how it influences response to direct KRAS G12C inhibitors will help enrich treatment populations and widen the therapeutic window. Ongoing investigation into rational combinations, both in the salvage setting and in the first-line setting, will increase the impact of both sotorasib and adagrasib in NSCLC and potentially in other tumours with KRAS G12C mutations. These agents will also influence biomarker testing, as it is now not only important to test for the presence of a KRAS mutation, but also to know which specific KRAS mutation is present.

### ***Resistance to KRAS G12C Inhibitors*** (Fig. 6)

**Fig. 6:**
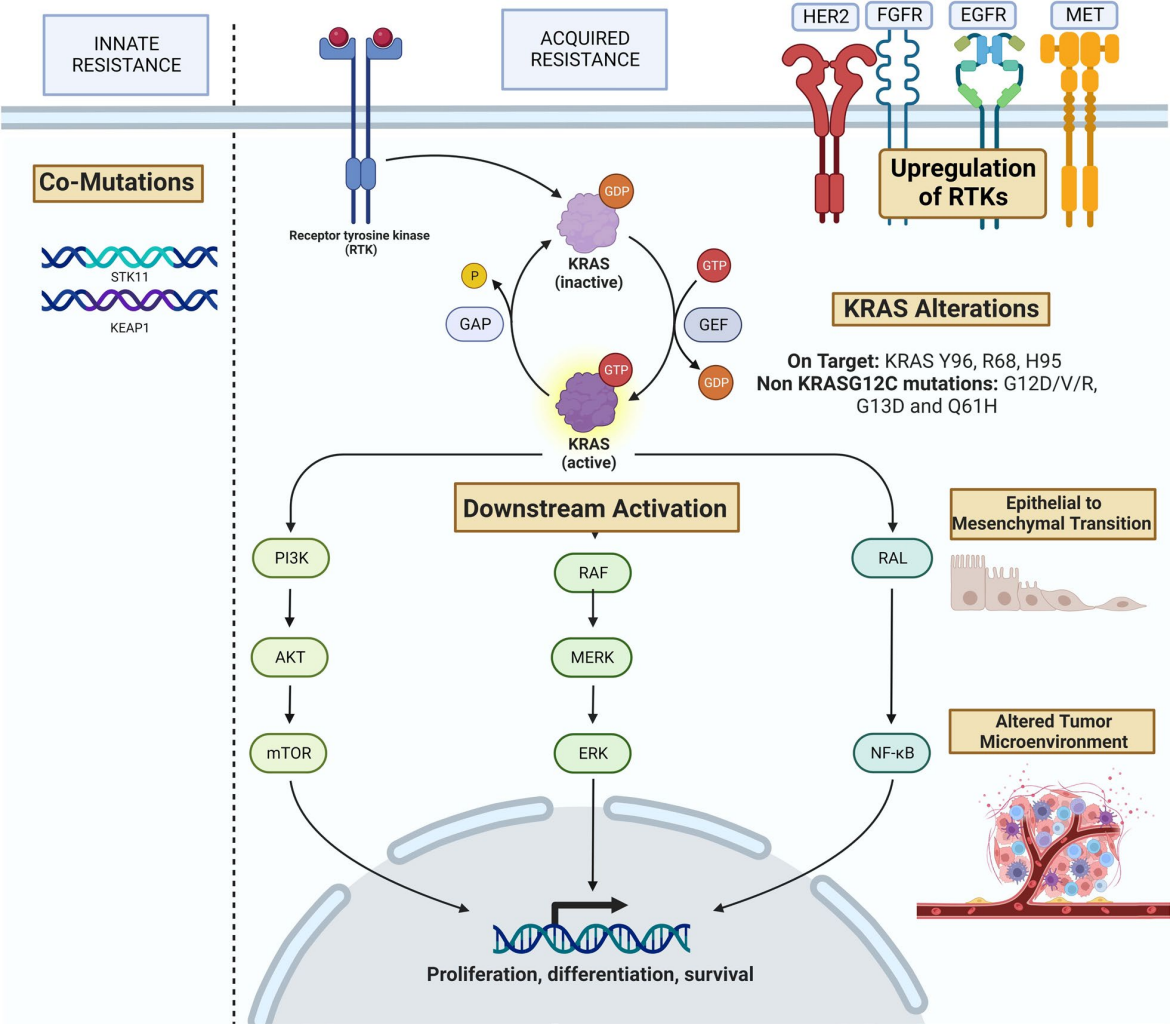
Resistance mechanisms to sotorasib and adagrasib

#### Innate resistance

In the phase 2 CodeBreaK100 and KRYSTAL-1 trials, while a majority of patients achieved disease control, less than 50% achieved any objective response. As such, it is important to try to understand the mechanisms of innate resistance, both from a scientific point of view and, ultimately, to further tailor molecular treatments. The heterogeneity of response among KRAS-mutant cancers is a far cry from those seen with other kinase inhibitors in NSCLC, for example. A possible explanation is that KRAS G12C mutation is more common among smokers, with roughly 93% of patients being current or former smokers in the latter trial, leading to a greater degree of molecular and genomic heterogeneity in these cancers. Tobacco-induced genomic alterations may offer alternative carcinogenic pathways despite the presence of a KRAS mutation [103]. Biomarkers such as PD-L1 and TMB, which have been studied to predict outcomes with immune checkpoint inhibitors, were not predictive or prognostic in patients with KRAS G12C-mutant NSCLC in CodeBreaK100 [104].

In the phase II portion of CodeBreaK100, responses to sotorasib were seen across PD-L1 strata [94]. The ORR in all PD-L1 evaluable patients (*n* = 86) was 42%, consistent with PD-L1 negative (48%) and PD-L1 low (39%). The ORR in PD-L1 high (≥ 50%) was 22%, but the sample size was limited (*n* = 9). Responses to adagrasib in the phase II portion of KRYSTAL-1 were similar across PD-L1 strata, with ORR 46.8% in PD-L1 negative, ORR 44.4% in PD-L1 low, and ORR 41.7% in PD-L1 high (≥ 50%). Responses to sotorasib did not vary between TMB high (40%) and TMB low (42%).

Concurrent mutations can also be associated with innate resistance to KRAS inhibition. In CodeBreak100 (phase II), among 104 patients whose tumours could be analyzed for co-mutations in STK11 and KEAP1, the ORR to sotorasib was 39%. While ORR was 50% in those with mutated STK11 and wild-type KEAP1, it was only 23% when both KEAP1 and STK11 were mutated and 14% when KEAP1 was mutated with wild-type STK11 (Table 3). In KRYSTAL-1, ORR to adagrasib in STK11 mutated with wild-type KEAP1 was 44.0%. When both KEAP1 and STK11 were mutated, ORR was 35.7% (5/14) and with KEAP1 mutated and wild-type STK11, ORR was 14.3% (1/7) (Table 3). On a biological level, cancers with KRAS G12C and STK11 co-mutations treated with adagrasib were associated with increased tumour infiltrating lymphocytes, suggesting that the drug alters the tumour microenvironment and reverses STK11-induced immunosuppression. STK11 and KEAP1 are tumour suppressor genes that appear to have a negative prognostic and perhaps predictive role in patients harbouring KRAS mutations and treated with immunotherapy [73].

**Table 3:** ORR in KRAS G12C NSCLC by STK11 and KEAP1 mutation status from single arm phase II studies of sotorasib (CodeBreaK100 [94]) and adagrasib (KRYSTAL-1 [100])

	Overall (%)	STK11mt, KEAP1wt (%)	STK11mt, KEAP1mt (%)	STK11wt, KEAP1mt (%)
Sotorasib 920 mg daily	37.1	50	23	14
Adagrasib 600 mg bid	42.9	44.0	35.7	14.3

At a preclinical level, primary resistance can result from a varied response in which some cells are eliminated by the kinase inhibitor while others synthesize new KRAS G12C, which is subsequently converted to an active, drug-refractory form [105]. Moreover, in CRISPR/Cas9-mediated KRAS knocked out PDAC cells, PI3K-driven MAPK activity was shown to promote tumourigenesis, independent of KRAS deficiency, showing potential for innate resistance to KRAS inhibitors [106]. A similar result was seen in a KRAS-mutant lung cancer cell line, wherein, the authors were able to classify KRAS-mutant cancer cells as KRAS-dependent or KRAS-independent [107]. A specific KRAS-dependent gene signature was associated with epithelial differentiation status. KRAS-independency was more likely to be associated with epithelial-to-mesenchymal transition (EMT), another mechanism of innate as well as acquired resistance to KRAS inhibition [108]. This mechanism can also explain non-KRAS driven tumourigenesis in which the KRAS mutation may act as a passenger alteration.

#### Acquired or adaptive resistance

In addition to better understanding and predicting initial response to selective KRAS G12C inhibitors, efforts to understand acquired resistance are also underway. Acquired resistance mechanisms can broadly be divided into three categories: (1) on-target resistance such as secondary or concurrent KRAS alterations, (2) off-target resistance such as upstream, downstream or parallel bypass mechanisms, (3) TME changes, and (4) histological transformation. Different resistance mechanisms have emerged, including concurrent KRAS alterations, vertical signaling alterations including RTK-RAS-MAPK, microenvironment changes and phenotypic transformation. In some cases, multiple resistance mechanisms emerge together [109].

##### Concurrent KRAS alterations

KRAS G12C mutations are not mutually exclusive with other KRAS alterations. Among KRAS-driven cancers, pooled analysis of multiple cancers shows that 2.8% of cases exhibit concurrent KRAS mutations [110]. A recent retrospective analysis of a large cohort of KRAS_mt_ NSCLC showed a concurrent KRAS mutation in 8% of patients with KRAS c.34G > T mutation. The most frequent concurrent mutation was KRAS c.35G > T, which if present in cis-position would lead to KRAS G12F protein and if present in trans-position, would lead to KRAS G12C and KRAS G12V proteins [111]. Cell lines with double-mutant KRAS G12C and G12V were found to be resistant to KRAS G12C inhibition in vitro. These escape mechanisms are found equally within the KRAS gene and downstream of KRAS [112]. As such, while the KRAS G12C signaling could remain blocked by small molecule kinase inhibitors, alternative KRAS activation pathways would be unimpeded, causing therapeutic resistance [110]. This is supported by the hypothesis that G12C inhibitors solely inhibit the inactive KRAS-GDP conformation, meaning that additional KRAS mutations leading to non-uniform cell cycling and preferential active KRAS-GTP conformation would be resistant to KRAS G12C inhibitors [105]. These on-target KRAS activating mutations can be classified into distinct classes. The first are switch-II binding pocket mutations, involving KRAS Y96, R68 and H95 residues. For instance, KRAS Y96D has been found to confer resistance to allele-specific KRAS G12C inhibitors by blocking their adhesion [109]. Another class of resistance is the result of activating non-G12C KRAS mutations, such as G12D/V/R, G13D and Q61H, often in a trans allelic configuration. Allelic rearrangement of G12C to G12W can also induce secondary drug resistance. Furthermore, there are specific mutations within the adagrasib binding pocket that can emerge, directly inhibiting the drug’s activity. KRAS G12C amplifications have also been detected as secondary on target alterations [112]. In an analysis of 38 patients with KRAS G12C cancers treated with adagrasib monotherapy, including 27 with NSCLC and 10 with colorectal cancer, mechanisms of acquired resistance were identified in 45% [113]. Of these, 41% (18% of the entire cohort) had multiple identified mechanisms. The most common alterations, representing 88% of resistance mechanisms, were associated with reactivation of the RAS-MAPK pathway including mutations in the adagrasib-binding pocket (KRAS Y96C, H95R, H95D, and R68S), and alternate KRAS activating mutations including KRAS G12D, G12V, and G13D (occurring in trans configuration with KRAS G12C on a separate allele). Interestingly, the presence of SIIP pocket mutations can vary between agents used, for example, H95 mutations were shown to decrease sensitivity to adagrasib but not sotorasib. In another study, 43 patients with KRAS G12C mutated cancers, including 36 NSCLC, were assessed in paired analyses upon progression on sotorasib [114]. 27 of 43 patients had treatment-emergent alterations with 4 of them having a secondary KRAS alteration and 3 having a low-level KRAS copy number gain.

##### Vertical signaling pathway alterations

Alterations impacting the RTK and other upstream signaling pathways of KRAS-GTP, including GRB2, SHP2 and SOS, can induce resistance to G12C inhibition. Similarly, numerous alterations converge on downstream MAPK signaling cascade activation which can impair kinase inhibitors. KRAS G12C inhibition induces concomitant suppression of DUSP, PHLDA and SPRY genes, which normally function as negative regulators of the MAPK pathway [115]. Furthermore, G12C inhibitors can increase MAPK signaling via upward regulation and phosphorylation of RTKs including EGFR, HER2, FGFR, ALK and MET caused by reactivated HRAS and NRAS signaling [116]. In fact, upstream RTK inhibition has demonstrated both in vitro and in vivo efficacy, restoring sensitivity to sotorasib in NSCLC.

Resistance pathways differ by tissue of tumour origin. In NSCLC, exposure to sotorasib causes upregulation of MEK and ERK which can lead to rapid resistance. In CRC, on the other hand, there is more frequent phosphorylation of EGFR, leading to the downstream MAPK cascade [117]. The latter mirrors EGFR-mediated resistance to BRAF inhibition in CRC and could explain the more modest efficacy of G12C inhibitor monotherapy compared to that found in NSCLC [118, 119].

In the studies comparing paired biopsies after emergence of resistance to sotorasib or adagrasib, alterations in NRAS, BRAF, EGFR, MET, were identified with both drugs, with additional FGFR2, MYC, IDH1/2 alterations among those treated with sotorasib [113, 114]. Much remains to be elucidated about the interplay of these alterations and the optimal approach to combine therapy, both to prevent and treat resistance mechanisms.

##### Other mechanisms

In addition to the vertical signaling cascades involved in resistance, parallel pathways can bypass KRAS G12C inhibition through a variety of mechanisms, including tumour microenvironment alterations, alterations in cell cycle regulators, and phenotypic transformation.

The tumour microenvironment changes under therapeutic pressure from sotorasib, with upregulated TGF-β signaling which can act as an upstream mediator of the MAPK cascade and favour EMT transformation, as well as complement activation, neoangiogenesis and coagulation [120]. The same study found that multiple gene signatures known to be associated with T and B cell immune function were downregulated upon resistance. In addition to these immune escape pathways, other microenvironmental changes include increased xenobiotic metabolism, which could allow the tumour to reduce intracellular sotorasib concentrations, rendering it ineffective. KRAS G12C inhibition also induces adhesion kinase activation, leading to fibrotic changes that may cause drug-resistance [121].

In NSCLC, roughly 20% of patients developed loss-of-function mutations in CDKN2A, a cell-cycle regulator. This deletion induces CDK4/6 RB phosphorylation and activation. In xenograft models, this cell-cycle dysregulation was reversed by adding a CDK4/6 inhibitor, palbociclib to adagrasib [91]. Another approach using the AURKA cell-cycle inhibitor, alisertib, could have similar effects [105].

Like with other targeted therapy, one off-target resistance mechanism is histologic transformation. EMT transformation is a known adaptive resistance mechanism both in EGFR and KRAS driven tumours, as described above. 2 patients among 38 receiving adagrasib developed a histologic transformation to squamous cell carcinoma [113]. Interestingly, no accompanying genomic changes were identified with this histologic transformation. No additional transformations have been identified to date, including to SCLC.

### Non-G12C KRAS inhibitors

While sotorasib is approved for KRAS G12C-mutant NSCLC, there remains a bigger unmet need of targeting other KRAS mutations, seen in higher frequency that G12C. A pertinent question is to ask is if the strategy for developing G12C inhibitors can be re-employed to target other KRAS mutations. Among these mutations, G12D is the commonest, followed by G12V, G12S, G12R and others. These mutant proteins lack an active residue such as cysteine and therefore, require a novel approach to noncovalently block the amino acid at codon 12. The second challenge with targeting G12X is that it lacks the intrinsic hydrolysis activity seen with G12C and is therefore, likely to remain in GTP-bound state [122]. This led to the development of BI-2852, a prototype in-vitro KRAS inhibitor that blocks both GDP-bound ‘OFF’ and GTP-bound ‘ON’ KRAS states [123]. Several ‘ON’ inhibitors of KRAS, which can be either selective for specific mutations or non-selective are now in development. ‘ON’ inhibitors are postulated to have significant advantages over ‘OFF’ inhibitors. These agents can lead to a much faster inhibition of RAS signaling, leading to quicker cell death and responses. They can be resistant to upstream RTK amplification, which is a known pathway of resistance to RAS inhibition.

Targeting KRAS G12C successfully opened the doors for developing newer agents to target other KRAS mutations directly. MRTX-1133 is a noncovalent KRAS G12D inhibitor that binds to the S-IIP of active as well as inactive states of KRAS G12D [124]. MRTX 1133 utilizes a piperazinyl group to form an ionic bond selectively with Asp12. It was shown to have in vivo activity and led to inhibition of ERK phosphorylation (pERK) as well as tumour regression in murine xenograft models. RMC-9805 is a KRAS G12D ‘ON’ inhibitor that blocks GTP-bound KRAS G12D using a novel tri-complex formation [125]. This agent uses cyclophilin A as an intracellular chaperone protein to form a non-covalent binary complex. The binary complex then binds to S-IIP of KRAS G12D-GTP complex to form a tri-complex of KRAS, cyclophilin-A and RMC-9805, and leads to covalent G12D cross-linkage, irreversibly blocking downstream effector binding to KRAS. Similar strategy is used by RMC-6291 and RMC-8839, selective KRAS G12C and G13C ‘ON’ inhibitors, respectively [126, 127]. A potential concern with these agents is inhibition of wild type KRAS and ensuing toxicities, however, preclinical data show limited wild type inhibition.

RMC-6236 is a non-selective RAS inhibitor that blocks interactions of multiple ‘ON’ RAS isoforms with downstream effectors using the above-mentioned tri-complex formation [128]. In preclinical models, RMC-6236 inhibited all RAS-mutant isoforms as well as wild type KRAS, HRAS and NRAS, however, inhibition was strongest for KRAS G12X-mutant cell lines. At oral doses of 25 mg/kg daily, RMC-6236 showed encouraging activity and durable responses in murine studies with KRAS G12X lung cancer, pancreatic cancer and colorectal cancer xenografts. Synergistic in vivo efficacy was also seen by combining RMC-6236 with anti-PD1 immune checkpoint inhibitor. Phase I clinical trial of RMC-6236 is currently ongoing (NCT5379985).

## Downstream signaling inhibition

Constitutively activated mutant KRAS renders upstream RTK inhibition ineffective. Several studies looked at downstream inhibition of the MAPK pathway in efforts to inhibit mutant KRAS signaling. Some initial promise of MEK inhibition either as monotherapy or in combination with chemotherapy was quickly found to be disappointing in clinical trials. Selumetinib did not show any PFS benefit over pemetrexed (67 days vs. 90 days, respectively; HR 1.08, *p* = 0.79) in patients with advanced NSCLC who received one or two lines of prior therapy [129]. Similarly, trametinib did not improve PFS and OS compared to docetaxel in a randomized trial of patients with KRAS-mutant NSCLC and the study was closed for futility [130]. Subsequently, combination studies were employed to evaluate any additive effects of MEK inhibition. A placebo-controlled phase II trial of selumetinib and docetaxel compared to docetaxel in KRAS-mutant NSCLC showed median OS of 5.2 and 9.4 months for selumetinib and placebo groups, respectively. It did show improved median PFS and ORR with the selumetinib and docetaxel combination of 5.3 months and 37% compared to 2.1 months (HR 0.58, *p* = 0.014) and 0% (*p* < 0.0001) for docetaxel and placebo [131]. This led to the phase III SELECT-1 trial, in which, 510 patients with KRAS-mutant NSCLC who had disease progression on one prior line of treatment were randomized to receive docetaxel with selumetinib or placebo. The study did not meet its primary endpoint of PFS. The median PFS for selumetinib and docetaxel was 3.9 months while that for placebo and docetaxel was 2.8 months (HR 0.93, 95% CI 0.77–1.12; *p* = 0.44). Median OS were similar between both groups but selumetinib was associated with a much higher rate of grade ≥ 3 adverse events (67% vs. 45%) [132]. Another small phase Ib study found no additional benefit of adding binimetinib to carboplatin and pemetrexed for frontline treatment of patients with KRAS-mutant NSCLC (PFS 5.7 months and OS 6.5 months) [133]. These results lead to the conclusion that downstream MEK inhibition without any KRAS inhibition is not a viable strategy for KRAS-mutant NSCLC.

The final enzyme in the MAPK pathway, cyclin D kinase, is a therapeutic target for malignancies, most notably breast cancer, with agents like palbociclib, ribociclib, and abemaciclib. Cdk4 gene ablation was associated with tumour death in genetically engineered KRAS-mutant NSCLC mouse models providing the preclinical rationale for CDK4 inhibition in NSCLC [134]. Human derived xenograft models showed abemaciclib was more effective against KRAS-mutant NSCLC than KRAS wild-type NSCLC. In a phase I study, abemaciclib showed 55% DCR and 2.8 months median PFS in KRAS-mutant NSCLC [135]. Subsequently, phase III JUNIPER trial randomized 453 patients with KRAS-mutant NSCLC who had disease progression on two prior lines of therapy to receive abemaciclib (*n* = 270) or erlotinib (*n* = 183) [136]. The study results were disappointing, and median OS was 7.4 months for abemaciclib and 7.8 months for erlotinib (HR 0.968, 95% CI 0.768–1.219; *p* = 0.77). Along similar lines, preclinical ERK inhibition of KRAS-mutant pancreatic cancer cell lines also showed MYC degradation and cellular suppression [137]. Clinical trials involving ERK inhibitors such as ulixertinib (BVD-523), temuterkib (LY3214996), rineterkib (LTT462), and JSI-1187 as monotherapy and in combination with other agents are ongoing in pancreatic, colorectal and non-small cell lung cancers [138–141]. There is now renewed interest in downstream pathway inhibition in combination with KRAS inhibitors.

In a combination arm of CodeBreaK101 trial of sotorasib and trametinib in KRAS G12C-mutant NSCLC and CRC, disease control rate was 67% and 86%, respectively, among patients with prior G12C inhibitor treatment [142]. However, 34% of patients had grade 3 or higher toxicity and 46% of patients required dose modification. TRAE leading to dose discontinuation was seen in 24% of patients.

Of note, KRAS G12V is being targeted, albeit indirectly, using a dual downstream blockade of RAF and MEK kinases. MEK inhibition has shown to paradoxically activate RAF-induced MEK phosphorylation by inhibiting ERK-dependent feedback loop [143]. VS-6766 is a dual RAF-MEK inhibitor which showed promising safety and efficacy in phase I basket trial of patients with solid malignancies or multiple myeloma who harboured mutations in the RAS-RAF-MEK pathway [144]. 7 out of 26 patients in the dose expansion phase had an objective response. 3 out of 10 patients with NSCLC had an objective response and 2 of those patients had KRAS G12V mutation. Focal adhesion kinase (FAK) induction was identified as a potential resistance mechanism to VS-6766. Therefore, a combination phase I trial of VS-6766 and FAK inhibitor, defactinib, in NSCLC was performed, which resulted in an ORR of 15% and again showed activity in KRAS G12V-mutant NSCLC (2 out of 2 responses) [145]. Phase I and phase II studies involving VS-6766 in combinations with defactinib (NCT04620330 and NCT04625270), everolimus (NCT02407509), cetuximab (NCT05200442), adagrasib (NCT05375994), and sotorasib (NCT05074810) are planned or underway in multiple malignancies including lung, ovarian, and colorectal cancers.

## Upstream signaling inhibition

### EGFR inhibitors

Activating RAS mutations are constitutively resistant to upstream EGFR or RTK blockade. A straightforward example of this is the indication of EGFR antibodies cetuximab and panitumumab only for KRAS wild type colorectal cancer. However, with the advent of KRAS inhibitors, there is some enthusiasm for dual RTK or EGFR blockade with KRAS inhibitors. A combination of adagrasib with cetuximab, an EGFR-directed monoclonal antibody, was explored in 32 patients with KRAS G12C colorectal cancer. The ORR with this combination was 43% with a disease control rate of 100% [102]. These results have led to opening of KRYSTAL-10, a phase III randomized trial of second line adagrasib and cetuximab compared with FOLFOX or FOLFIRI in patients with KRAS G12C-mutant colorectal cancer (NCT04793958). Similarly, sotorasib and panitumumab combination in CodeBreaK101 study also showed an ORR of 16.6% and DCR of 83.3% in KRAS G12C-mutant colorectal cancer [146]. In another arm of CodeBreaK101 trial, pan-ErBB inhibition with afatinib combined with sotorasib in 33 patients with KRAS G12C-mutant malignancies showed an ORR of 30.3% and DCR of 75.8% [147]. However, this was also a relatively toxic combination with 35% of patients experiencing grade 3 or higher TRAEs.

### SOS1 inhibitors

As RAS shuttles between ‘ON’ and ‘OFF’ states, it is vulnerable to deactivation by inhibiting its upstream GEF, SOS1. SOS1 inhibition can effectively inhibit RAS-GTP binding and there are multiple SOS1 inhibitors in development. BAY-293 was the first SOS1 inhibitor that showed decreased RAS-mediated signaling in KRAS wild type and G12C-mutant cell lines [148]. The combination of ARS-853 and BAY-293 also showed synergistic antiproliferative activity in a KRAS G12C-mutant cell line. BI-3406, another tool compound targeting SOS1 was shown to have in vitro activity in KRAS G12X-mutant cell lines [149]. This compound selectively inhibited SOS1 and decreased pERK in several G12 and G13 variants, but not in KRAS Q61H and G12R variants, which are known to lack intrinsic hydrolysis and SOS1 catalytic domain interaction, respectively. SOS1 reactivation is known to be a resistance mechanism to MEK inhibition [150]. BI-3406 inhibited feedback reactivation induced by trametinib, guiding the rationale of concurrent SOS1 and MEK inhibition. BI 1701963, the clinical analogue of BI-3406, showed a tolerable safety profile in as a single agent, with grade 3 or higher TRAEs included hypertension, congestive cardiomyopathy and decreased platelet count [151]. It is also being studied in combinations with adagrasib (NCT04975256) and trametinib (NCT04111458). MRTX0902 is another SOS1 inhibitor that showed in vitro and in vivo efficacy when combined with MRTX849 in KRAS G12C-mutant NSCLC models compared to MRTX849 alone [152].

### SHP2 inhibitors

SHP2 activation by RTKs promotes GEF-mediated RAS-GTP interaction and activates RAS-RAF-ERK pathway. SHP099 was the first orally bioavailable allosteric SHP2 inhibitor that demonstrated in vivo inhibitory activity in RTK-driven xenograft models [153]. Subsequently, multiple studies showed that SHP2 inhibition prevented adaptive resistance and synergized with MEK inhibitors in multiple preclinical KRAS-mutant cancer models [154–156]. Interestingly, SHP2-mediated signaling was found to be necessary for KRAS G12C driven oncogenesis, paving the rationale for combining SHP2 and KRAS inhibitors [157]. Furthermore, as SHP2 inhibition prevents KRAS-GTP binding, it may potentiate covalent KRAS G12C inhibitors. The combination of a preclinical tool SHP2 inhibitor RMC-4550 and MRTX849 led to greater decrease in pERK compared to MRTX849 alone in KRAS G12C-mutant xenograft models [91]. Along similar lines, combinations of TNO155 with EGFR inhibitors, BRAF and MEK inhibitors, and CDK4 inhibitor, ribociclib showed synergistic preclinical activity [158]. Several SHP2 inhibitors including TNO155, RMC-4630, RLY-1971 and JAB-3068 are currently under evaluation as single agents as well as in combinations with KRAS and other MAPK pathway inhibitors. RMC-4630 monotherapy was found to be well tolerated and was associated with a disease control rate of 71% (5/7) in NSCLC [159]. The combination of RMC-4630 and sotorasib was also found to be well tolerated with grade 1 or 2 edema as the most frequent treatment related adverse event (30%) and led to a disease control rate of 64% in patients with NSCLC who were previously treated with a KRAS G12C inhibitor. In KRAS G12C inhibitor-naïve patients, DCR was 100% and ORR was 50% [160]. As SHP2 inhibition is not subject to MAPK pathway mutations alone, there is a theoretical concern for toxicity, that may hinder development of rationally sound combinations.

## Combinations with immunotherapy

As we described earlier, KRAS and its co-mutational status can alter the immune microenvironment and render it immunosuppressive. KRAS G12C inhibitors sotorasib and adagrasib have shown to promote a pro-inflammatory microenvironment in murine models by increasing CD8 + T-cell infiltration and by decreasing immunosuppressive cells such as MDSCs, M2-polarized macrophages, Tregs and potentiate anti-PD1 antibody therapy [71, 161]. Recently, KRAS G12C inhibition was shown to upregulate interferon signaling, enhanced CD8 + T-cell infiltration and synergism with immune checkpoint inhibition in immunogenic murine lung cancer models. However, KRAS G12C inhibition did not sensitize non-immunogenic murine models to immunotherapy. This preclinical finding may guide in selecting patients for combinational clinical trials [162]. Combinations of G12C inhibitors and immune checkpoint inhibitors are being studied in frontline and refractory settings in NSCLC (NCT04613596, NCT04185883, NCT03600883). The CodeBreaK 100/101 study, which reported the safety and efficacy of sotorasib in combination with pembrolizumab or atezolizumab showed grade 3 or higher hepatotoxicity in 9 out of 19 patients treated concurrently with sotorasib and pembrolizumab [163]. Another recent case report of severe immune related hepatitis with sotorasib in a patient previously treated with pembrolizumab also points at persistent risk of hepatotoxicity with sequencing immunotherapy followed by sotorasib [164]. It is now known that PD1-PDL1 binding recruits SHP2 to assist in forming negative costimulatory microclusters that inhibit T-cell receptor signaling [165]. This suggests that SHP2 inhibition can inhibit PD1-PDL1 downstream signaling and may potentiate anti-PD1/PDL1 agents. Accordingly, SHP2 inhibition was also shown to promote T-cell proliferation and tumour cell killing in cell cultures. The combination of SHP2 inhibitor and anti-PD1 antibody has also shown to CD8 + T cell recruitment, a decrease in myeloid derived suppressive cells, and heightened anti-tumour immunity in mouse models [166, 167]. TNO155 is being studied in combination with anti-PD1 antibody spartalizumab in various malignancies (NCT04000529). As we await results of studies using KRAS inhibitors in the first line of treatment, immunotherapy and combination of chemotherapy and immunotherapy remain the standard of care for patients with metastatic KRAS-mutant NSCLC.

## Future perspectives

The FDA approval of the first mutant selective KRAS G12C inhibitor is the fruit of decades of research and KRAS drug development. This is just the beginning as we are seeing the tip of the iceberg and we look forward for hundreds of drugs being developed in this space. Better understanding of structural elements of KRAS and KRAS mutants combined with novel, computerized drug screening technologies have increased our ability of potent and selective drug development. For the near future, the challenge is to better understand resistance mechanisms to G12C inhibitors, develop strategies to overcome on- and off-target resistance, and effectively target other commonly seen KRAS mutations. A noteworthy recent breakthrough is the chemical development of selective covalent ligands for the mutant arginine residue in KRAS G12R and mutant serine residue in KRAS G12S [168, 169]. Combination therapies of KRAS inhibitors with other inhibitors, cytotoxic chemotherapy agents and immunotherapies are generating excitement, but we suggest cautious optimism and phase III evidence to define the effectiveness of these agents and their combinations. While a detailed discussion on new treatments such as vaccine therapies, cellular therapies, and protein degraders is beyond the scope of this review, this space will need to be watched closely in the future. Peptide vaccines targeting KRAS have been tried, albeit with limited success, likely owing to limited generation of epitopes for immune recognition [170, 171]. Newer vaccine studies featuring personalized dendritic cell vaccine, MIDRIX4-LUNG (NCT03592888), mRNA vaccines, mRNA-4157 (NCT03313778) and V491 (NCT03948763) are ongoing and results are awaited. Proteolytic targeted chimeras (PROTACs) are protein degraders that facilitate ubiquitin-proteosome system-based degradation of a target protein. A PROTAC consists of a ligand to the ubiquitinase E3, a ligand (small molecule inhibitor) to the protein of interest (POI) and a linker. A KRAS G12C PROTAC, LC2, developed with MRTX849 ligand was found to successfully degrade KRAS G12C in cancer cells, but limited by its covalent nature of binding and limited potency [172]. Newer PROTACs with catalytic activity are currently being developed. In conclusion, development of therapeutically active allosteric KRAS inhibitors has ushered a new era of therapies to target an old foe. Over time, we are likely to see several broad and personalized therapies for patients with KRAS-mutant malignancies.

## Data Availability

Not applicable.
